# Supplementary material for: HIVprotI: an integrated web based platform for prediction and design of HIV proteins inhibitors
Source: J Cheminform. 2018 Mar 9;10:12. doi: 10.1186/s13321-018-0266-y (PMC5845081; doi:10.1186/s13321-018-0266-y)
Supplement: Supplementary file 2 — Additional file 2. Source code of HIVProtI web server. [file 13321_2018_266_MOESM2_ESM.zip › HIVprotI_Source-code/hivprotI/weka-3-6-6/doc/weka/experiment/RemoteExperimentSubTask.html]

RemoteExperimentSubTask


---


|  |  |  |
| --- | --- | --- |
| **Overview** \| **Package** \| **Class** \| **Tree** \| **Deprecated** \| **Index** \| **Help** \| **Weka's home** | |  |
| **PREV CLASS**   **NEXT CLASS** | **FRAMES**    **NO FRAMES**     **All Classes** |  |
| SUMMARY: NESTED \| FIELD \| CONSTR \| METHOD | DETAIL: FIELD \| CONSTR \| METHOD |  |


---


## weka.experiment Class RemoteExperimentSubTask

```
java.lang.Object
  weka.experiment.RemoteExperimentSubTask
```

**All Implemented Interfaces:**: java.io.Serializable, RevisionHandler, Task

---

``` public class RemoteExperimentSubTask extends java.lang.Object implements Task, RevisionHandler ```

Class to encapsulate an experiment as a task that can be executed on
a remote host.

**Version:**
:   $Revision: 1.10 $

**Author:**
:   Mark Hall (mhall@cs.waikato.ac.nz)

**See Also:**: Serialized Form

---

**Constructor Summary**

`RemoteExperimentSubTask()`


| **Method Summary** | |
| --- | --- |
| `void` | `execute()`             Run the experiment |
| `Experiment` | `getExperiment()`             Get the experiment for this sub task |
| `java.lang.String` | `getRevision()`             Returns the revision string. |
| `TaskStatusInfo` | `getTaskStatus()`             Clients should be able to call this method at any time to obtain information on a current task. |
| `void` | `setExperiment(Experiment task)`             Set the experiment for this sub task |

**Methods inherited from class java.lang.Object**

`equals, getClass, hashCode, notify, notifyAll, toString, wait, wait, wait`

**Constructor Detail**

### RemoteExperimentSubTask

```
public RemoteExperimentSubTask()
```


**Method Detail**

### setExperiment

```
public void setExperiment(Experiment task)
```

:   Set the experiment for this sub task

    :   **Parameters:**: `task` - the experiment

---


### getExperiment

```
public Experiment getExperiment()
```

:   Get the experiment for this sub task

    :   **Returns:**: this sub task's experiment

---


### execute

```
public void execute()
```

:   Run the experiment

    :   **Specified by:**: `execute` in interface `Task`

---


### getTaskStatus

```
public TaskStatusInfo getTaskStatus()
```

:   **Description copied from interface: `Task`**
:   Clients should be able to call this method at any time to obtain
    information on a current task.

    :   **Specified by:**: `getTaskStatus` in interface `Task`
    :   **Returns:**: a TaskStatusInfo object holding info and result (if available) for this task

---


### getRevision

```
public java.lang.String getRevision()
```

:   Returns the revision string.

    :   **Specified by:**: `getRevision` in interface `RevisionHandler`
    :   **Returns:**: the revision


---


|  |  |  |
| --- | --- | --- |
| **Overview** \| **Package** \| **Class** \| **Tree** \| **Deprecated** \| **Index** \| **Help** \| **Weka's home** | |  |
| **PREV CLASS**   **NEXT CLASS** | **FRAMES**    **NO FRAMES**     **All Classes** |  |
| SUMMARY: NESTED \| FIELD \| CONSTR \| METHOD | DETAIL: FIELD \| CONSTR \| METHOD |  |


---
